# Significant Association of Cognitive Function With Long-Term Clinical Outcomes After Transcatheter Aortic Valve Implantation: A Retrospective Cohort Study

**DOI:** 10.7759/cureus.76045

**Published:** 2024-12-19

**Authors:** Tohru Kaga, Itsuka Kaga, Shinichiro Ueda

**Affiliations:** 1 Department of Clinical Research and Quality Management, Graduate School of Medicine, University of the Ryukyus, Okinawa, JPN; 2 Department of Psychiatry, Gunma Prefectural Psychiatric Medical Center, Gunma, JPN

**Keywords:** cognitive function, long-term outcome, mini-mental state examination, preoperative assessment and risk management, transcatheter aortic valve implantation

## Abstract

Background

Patients undergoing transcatheter aortic valve implantation (TAVI) are often elderly, and perioperative and long-term risk assessments should primarily consider cognitive function, comorbidities, and procedural complexity. This study investigated the association between cognitive function and mortality in patients with severe aortic valve stenosis (AS) who underwent TAVI.

Methodology

This single-center, retrospective cohort study consecutively registered patients who underwent TAVI between December 2014 and December 2018. We divided the patients into two groups using a Mini-Mental State Examination (MMSE) score of 23. All-cause mortality was the primary outcome of this study.

Results

The study cohort included 114 patients with a follow-up period of five years. The mean age of the patients was 85 years. Overall, 37 of the 104 patients who underwent preoperative MMSE tests were considered to have cognitive impairment. The risk of all-cause death after TAVI was significantly higher in patients with impaired cognitive function than in those with preserved cognitive function (hazard ratio = 4.27; 95% confidence interval = 1.90-9.57). However, there were no significant differences in the function of the left ventricle and prosthetic aortic valve between the groups.

Conclusions

Impaired cognition significantly and independently affected the long-term outcomes of patients with severe AS who underwent TAVI. Given the age of the patients in this study, the indications for TAVI should be carefully considered for each patient based on their cognitive function.

## Introduction

Since its introduction, transcatheter aortic valve implantation (TAVI) has become popular and has replaced surgical aortic valve replacement (SAVR). Initially, TAVI was a choice for elderly or high-risk patients but recently it became feasible for younger patients because its outcome in low-risk patients has been shown to be superior to that of SAVR, with valve durability not inferior to that of SAVR [1,2]. The Society of Thoracic Surgeons’ score [3] and Euroscore [4] are widely used to calculate the short-term outcomes of cardiac surgery. These scores focus on comorbidities, pathology of heart diseases, and details of the procedure but do not include patients’ functional characteristics such as independence or cognitive function. However, recent studies indicate that preoperative frailty and cognitive function significantly affect mortality and other adverse outcomes after TAVI during hospital stay and for a relatively short period after discharge [5-10]. However, the long-term outcomes of TAVI associated with cognitive status have not been reported.

According to a report by the Japanese Ministry of Health, Labour and Welfare in 2017, an 85-year-old man was expected to live for 6.26 years, and a similarly aged woman was expected to live 8.39 years. Although TAVI is much less invasive than SAVR, complications requiring surgical intervention, such as aortic annulus rupture, coronary artery occlusion, and access route injury, can occur. Patients undergoing TAVI may not tolerate surgical conversion. If TAVI does not improve prognosis at any age, the indications for TAVI may require reconsideration.

Therefore, we conducted a retrospective cohort study to explore the association between cognitive function and mortality in patients with severe aortic valve stenosis (AS) who underwent TAVI.

## Materials and methods

Study design

We conducted a single-center, retrospective cohort study of patients with severe AS who underwent TAVI at our hospital. The index date was defined as the date of TAVI performed between December 2014 and December 2018. Patients were divided into two groups based on Mini-Mental State Examination (MMSE) scores (Group 1 ≥ 24, Group 2 ≤ 23).

Study setting

This study was conducted at the Gunma Prefectural Cardiovascular Center. This facility specializes in cardiac and vascular diseases and is the only leading high-volume center in Gunma prefecture.

Participants

The patients were diagnosed with severe AS and were referred to a structural cardiology specialist. Age, physical status, independence, and comorbidities were reviewed and cardiologists assessed whether patients preferred SAVR. To assess patients’ independence, basic activities of daily living (ADLs) were assessed in the cardiologists’ clinic, which included walking, feeding, dressing/grooming, toileting, bathing, and transferring. Patients who could manage these activities could be listed at the conference. Patients considered to be at high risk for SAVR were assigned to a multidisciplinary team. Before accepting patients for TAVI, candidates were discussed at a heart team conference that included interventional cardiologists, cardiac surgeons, anesthetists, and perfusionists. For as long as possible, the patients’ MMSE scores were obtained by physiotherapists.

Inclusion and exclusion criteria

A total of 138 patients who underwent TAVI between December 2014 and December 2018 were recruited into this study. Among 138 patients, 34 patients who did not complete the preoperative MMSE test were excluded. Overall, 17 of 104 patients who had preoperative MMSE scores but could not be followed up at five years were excluded. Eventually, 87 patients with preoperative MMSE scores who completed the five-year follow-up were included in the study.

Ethical approval

The ethics committee of the Gunma Prefectural Cardiovascular Center approved this study (approval code: 2021008) following the Ethical Guidelines for Medical and Health Research Involving Human Subjects in Japan.

Consent

The requirement for written informed consent was waived before surgery. The surgery consent form explained that the patient’s data could be used for research while being anonymized. Patients or their families could request withdrawal from the study if they are unwilling to participate.

Measurements of preoperative cognitive function

The MMSE score, which is a global assessment of cognitive status on a scale of 0-30, was measured preoperatively by physiotherapists. A score ≤23 indicated dementia [11].

Measurement of left ventricular and prosthetic aortic valve function

Transthoracic echocardiography (TTE) was performed preoperatively and annually after TAVI to assess the function of the left ventricle and prosthetic aortic valve, including ejection fraction (EF, %), aortic valve area (AoVA, cm^2^), effective orifice area (EOA, cm^2^) of the prosthetic aortic valve, transvalvular jet velocity (m/s), transvalvular median, and peak pressure gradient (mmHg).

Outcomes

The primary study outcome was all-cause mortality at five years. The secondary outcome was rehospitalization at any hospital.

Follow-up

Most patients underwent regular echocardiography annually. Some patients could not undergo regular check-ups because they lived far from the hospital or had difficulty reaching the hospital owing to reduced physical activity. We called these patients to ask about their survival and the incidence of complications.

Statistical analysis

Descriptive Statistics

The clinical characteristics of the patients were described and compared. Continuous variables are expressed as mean ± standard deviation (SD) or median and interquartile range (IQR) according to their distribution. Comparisons were made using the Student’s t-test or Wilcoxon rank-sum test. Categorical variables are expressed as values and percentages and were assessed using the chi-square or Fisher’s exact test. The incidence rate of all-cause death and rehospitalization was described as 100 person-years.

Survival Analysis

We used the log-rank test to evaluate the survival probabilities and the Cox hazard ratio model to estimate the hazard ratio of mortality adjusted by age in the impaired cognitive status group compared to the intact cognitive status group. Statistical significance was set at p-values <0.05.

Comparison of Left Ventricular and Prosthetic Aortic Valve Function

Repeated-measures analysis of variance (ANOVA) was used to analyze continuous variables (EF, AoVA, EOA, transvalvular jet velocity, transvalvular median, and peak pressure gradient). Paravalvular leakage was classified into two groups (non-trivial to mild and moderate to severe), considering that more than moderate regurgitation was considered clinically significant. Differences in this variable were assessed using the chi-square test or Fisher’s exact test.

## Results

Patient characteristics

**Figure 1:**
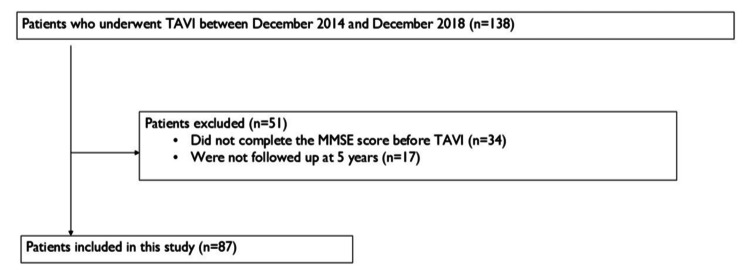
Flow profile and exclusion criteria for selecting the study population. TAVI: transcatheter aortic valve implantation; MMSE: Mini-Mental State Examination

A total of 138 patients underwent TAVI between December 2014 and December 2018. We could follow up 114 patients for five years, and 104 had preoperative MMSE scores. Among these 104 patients, 17 patients who we could not follow up at five years were excluded. Hence, 87 patients with preoperative MMSE scores who completed the five-year follow-up were included in the study (Figure 1). Table 1 shows the well-balanced characteristics of the two groups. Except for a history of percutaneous coronary intervention, there were no significant differences in background data. The median patient age was 85 years (IQR = 81-88), and 65.9% of the patients were women.

**Table 1:** Baseline characteristics of the included patients. BMI: body mass index; NYHA: New York Heart Association; MMSE: Mini-Mental State Examination; HTN: hypertension; DM: diabetes mellitus; HL: hyperlipidemia; HUA: hyperuricemia; AoVA: aortic valve area; CAD: coronary artery disease; CVD: cerebrovascular disease; PVD: peripheral vascular disease; CKD: chronic kidney disease; eGFR: estimated glomerular filtration rate; COPD: chronic obstructive pulmonary disease; AF: atrial fibrillation; MR: mitral regurgitation; MI: myocardial infarction; PCI: percutaneous coronary intervention; CABG: coronary artery bypass grafting; EF: ejection fraction; AoVA: aortic valve area; PG: pressure gradient; BAV: bicuspid aortic valve *: Wilcoxon test; **: t-test; ***: Fisher’s χ^2^ test

	Overall	Group 1 (MMSE ≥ 24)	Group 2 (MMSE ≤ 23)	P-value
n	138	67	37	
Sex (M/F)	47/91	27/40	10/27	0.2172^**^
Age (years)	85 (81–88)	84 (81–87)	86 (82.5–90)	0.2506^*^
BMI (kg/m^2^)	21.43 (19.29–24.17)	21.43 (19.36–24.31)	20.93 (18.78–23.39)	0.3626^*^
Preoperative NYHA (%) (I/II/III/IV)	10.4/41.5/42.5/5.6	13.4/44.8/37.3/4.5	3.0/38.2/50/8.8	0.2929^***^
Preoperative MMSE	25.5 (23–28)	27 (26–29)	21 (17–23)	<0.0001^*^
HTN	89	42	23	0.1492^**^
DM	40	21	9	0.3232^**^
HL	53	24	17	0.0919^**^
HUA	31	13	9	0.9650^**^
CAD	60	27	18	0.2192^**^
CVD	19	8	6	0.2476^**^
PVD	6	1	4	0.3573^**^
CKD	55	24	13	0.4545^**^
Creatinine (mg/dL)	0.89 (0.74–1.19)	0.88 (0.70–1.16)	0.82 (0.68–1.15)	0.3525^*^
eGFR (mL/min/1.73 m^2^)	51 (36.75–66)	53 (40–66)	51 (42–70)	0.2957^*^
Smoking (ex-smoker/current smoker)	41/3	21/1	7/2	0.8247^***^
COPD	9	3	4	0.1984^**^
Asthma	7	3	2	0.8671^**^
Heart failure	50	27	11	0.212^**^
Chronic AF	26	16	6	0.2366^**^
Moderate to severe MR	4	1	1	0.4341^**^
Previous MI	11	6	4	0.5847^**^
History of PCI	39	17	14	0.0137^**^
History of CABG	14	7	5	0.8665^**^
Preoperative EF	65 (60–70)	65 (55–70)	65 (57.5–70)	0.9065^*^
Preoperative AoVA (cm^2^)	0.65 (0.5275–0.78)	0.65 (0.5325–0.78)	0.61 (0.44–0.74)	0.1613^*^
Preoperative mean PG (mmHg)	49 (40–65.25)	49.5 (41–65)	56 (43–72)	0.9723^*^
Preoperative peak PG (mmHg)	80 (65.75–102)	82.5 (60.1–102)	90 (68–116)	0.9908^*^
Preoperative velocity (m)	4.5 (4–5.1)	4.6 (4.1–5.1)	4.8 (4.1–5.4)	1.0000^*^
BAV (bicuspid aortic valve)	2	2	0	0.4341^**^
Previous open-heart surgery	24	13	6	0.6872^**^
Liver disease	12	7	2	0.3727^**^
Albumin (g/dL)	3.95 (3.6–4.2)	3.9 (3.5–4.2)	4 (3.7–4.3)	0.2045^*^
Hematology disease	7	0	3	0.6900^**^
Cancer	26	11	8	0.8168^**^
Thyroid disease	5	4	0	0.2639^**^
Dementia	10	4	0	0.2639^**^
Psychotic disorder	1	1	0	0.5821^**^

Procedural characteristics

All TAVI procedures were performed under general anesthesia. The procedural characteristics are shown in Table 2. TAVI was performed via different routes: transfemoral, 131 (95%), trans-subclavian 1, transiliac artery 1, trans-apex 4, and direct aortic access, 1. Five prosthetic valves were used: Sapien XT and Sapien 3 (Edwards Lifesciences, Irvine, CA, USA), as well as CoreValve, Evolute R, and Evolute Pro (Medtronic, Minneapolis, MN, USA). The proportion of prosthetic valves was Sapien XT, 44 (31.9%); Sapien 3, 73 (52.9%); Medtronic Core Valve, 7 (5.1%); Evolut R, 12 (8.7%); and Evolut Pro, 2 (1.4%).

**Table 2:** Characteristics of TAVI. TAVI: transcatheter aortic valve implantation

Group		Overall	Group 1	Group 2
n		138	67	37
Access route	Femoral	131	66	34
	Apex	4	0	1
	Direct aorta	1	0	1
	Subclavian	1	0	1
	Iliac artery	1	1	0
Name of prosthetic valve	SAPIEN XT	44	21	11
	SAPIEN 3	73	34	19
	Core Valve	7	2	4
	Evolute R	12	9	2
	Evolute Pro	2	1	1
Required access route change		1	1	0
Conversion to surgery		12	5	2
Aortic reintervention		4	1	1

One patient required an access route change, and 12 patients underwent conversion to open surgery due to access route injury or aortic root rupture. In total, 13 patients had a stroke or transient ischemic attack intraoperatively or postoperatively, and nine of these patients had a disabling stroke.

All-cause death and cognitive function

**Figure 2:**
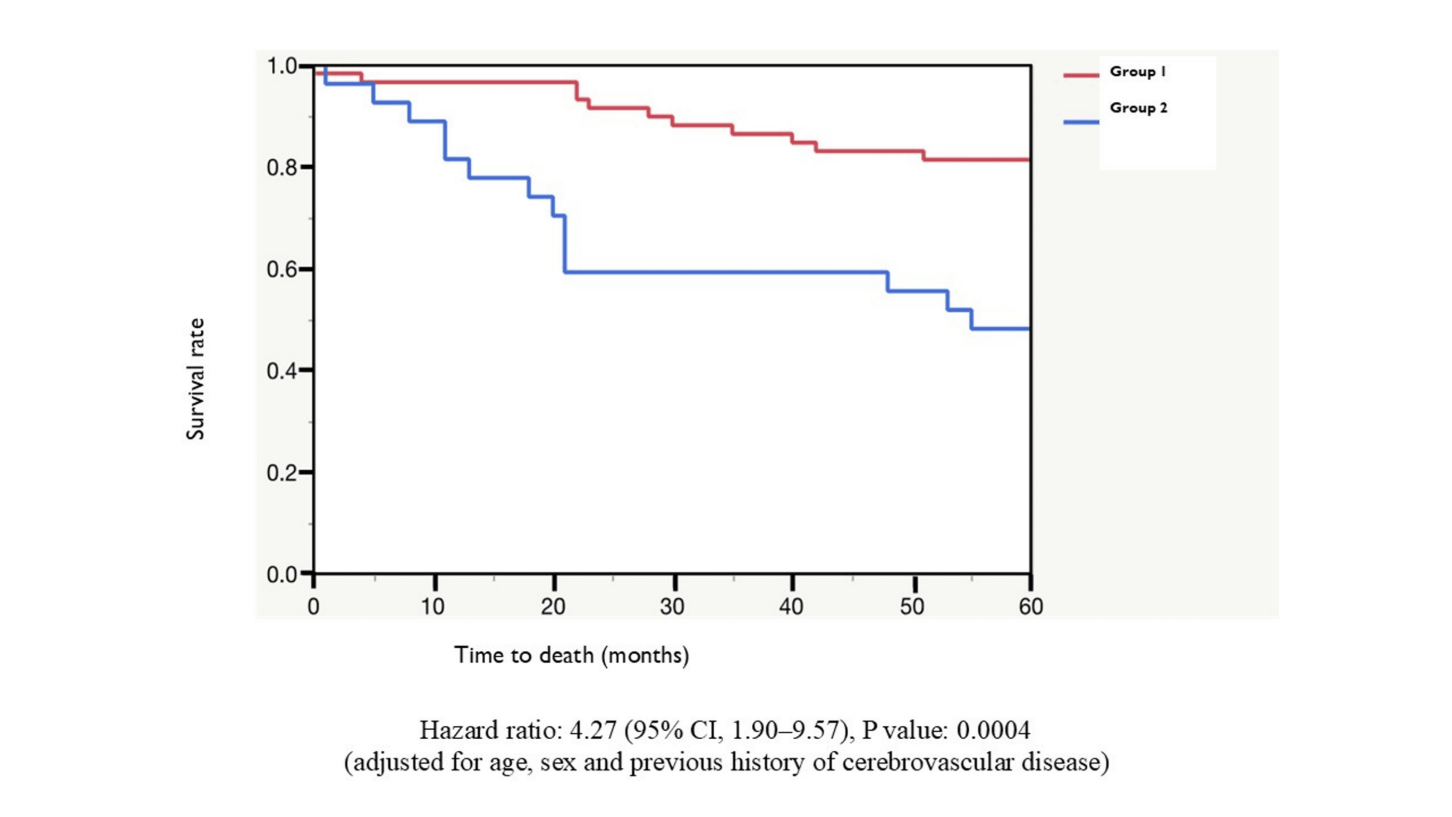
Kaplan-Meier survival curve. Group 1 (MMSE ≥ 24) had a shorter survival time compared to Group 2 (MMSE ≤ 23). Hazard ratio = 4.27; 95% confidence interval = 1.90 to 9.57, p = 0.0004 (adjusted for age, sex, and history of stroke). MMSE: Mini-Mental State Examination

Overall, 76 patients survived for five years, and 38 died within five years after TAVI. The incidence rate (Table 3) was 8.49 per 100 patient-years. In Group 1, 47 patients survived for five years, and 13 died (incidence rate, 4.91 per 100 patient-years). In Group 2, 13 patients survived for five years, and 14 died (incidence rate, 15.47 per 100 patient-years). Kaplan-Meier survival curves (Figure 2) showed that there was no significant difference in the short-term outcome until five months after TAVI between the two groups, but a significant difference was observed thereafter. The adjusted hazard ratio (HR), adjusted for age, sex, and previous history of cerebrovascular diseases, was 4.27 (95% confidence interval (CI) = 1.90-9.57). The cause of death (Table 4) was cardiac (n = 4), non-cardiac (n = 23), and unknown (n = 11) overall (cardiac, n = 1; non-cardiac, n = 10; unknown, n = 2 in Group 1; cardiac, n = 1; non-cardiac, n = 7; unknown, n = 6; in Group 2).

**Table 3:** Incident rate of mortality and rehospitalization.

	Death	Person-years	Incident rate per 100 persons
Overall	38	447.58	8.49
Group 1	13	264.75	4.91
Group 2	14	90.5	15.47
	Rehospitalization	Person-years	Incident rate per 100 persons
Overall	70	447.58	15.64
Group 1	34	264.75	12.84
Group 2	19	90.5	20.99

**Table 4:** Outcomes: survival, rehospitalization, and complications. CAG: coronary angiography; PCI: percutaneous coronary intervention; PMI: pacemaker insertion; PM: pacemaker

Group (n)		Overall (114)	Group 1 (60)	Group 2 (27)
Alive		76	47	13
Dead		38	13	14
Cause of death	Cardiac	4	1	1
	Non-cardiac	23	10	7
	Unknown	11	2	6
Repeat hospitalization	Catheter intervention (aortic reintervention, CAG, PCI)	4	3	1
	Non-valve surgery (PMI, PM lead removal, orthopedic)	6	6	0
	Heart failure	4	3	1
	Non-cardiac	56	22	17
Major bleeding		6	2	1
Major vascular complication		12	7	2
Renal failure	Total	21	12	3
	New hemodialysis	1	1	0
Stroke or transient ischemic attack		13	7	3
Stroke with disability		9	3	3
Structural valve deterioration		0	0	0
Permanent pacemaker implantation		16	11	2
Endocarditis	1	1 (mitral valve)	0	
Myocardial infarction	0	0	0	

Rehospitalizations and complications

Overall, 70 patients were readmitted within five years after TAVI (Table 3), with an incidence rate (Table 4) of 15.64 per 100 patient-years, as follows: four patients for catheter intervention, six for non-valve-related surgery, four for heart failure, and 56 for non-cardiac reasons. In Group 1, 34 patients were readmitted five years after TAVI, with an incident rate of 12.84 per 100 patient-years, as follows: three patients for catheter intervention, six for non-valve-related surgery, three for heart failure, and 22 for non-cardiac reasons. In Group 2, 19 patients were readmitted within five years post-TAVI, with an incident rate of 20.99 per 100 patient-years, as follows: one patient for catheter intervention, one for heart failure, and 17 for non-cardiac reasons. The incidences of postoperative complications are shown in Table 3.

Echocardiography findings

**Figure 3:**
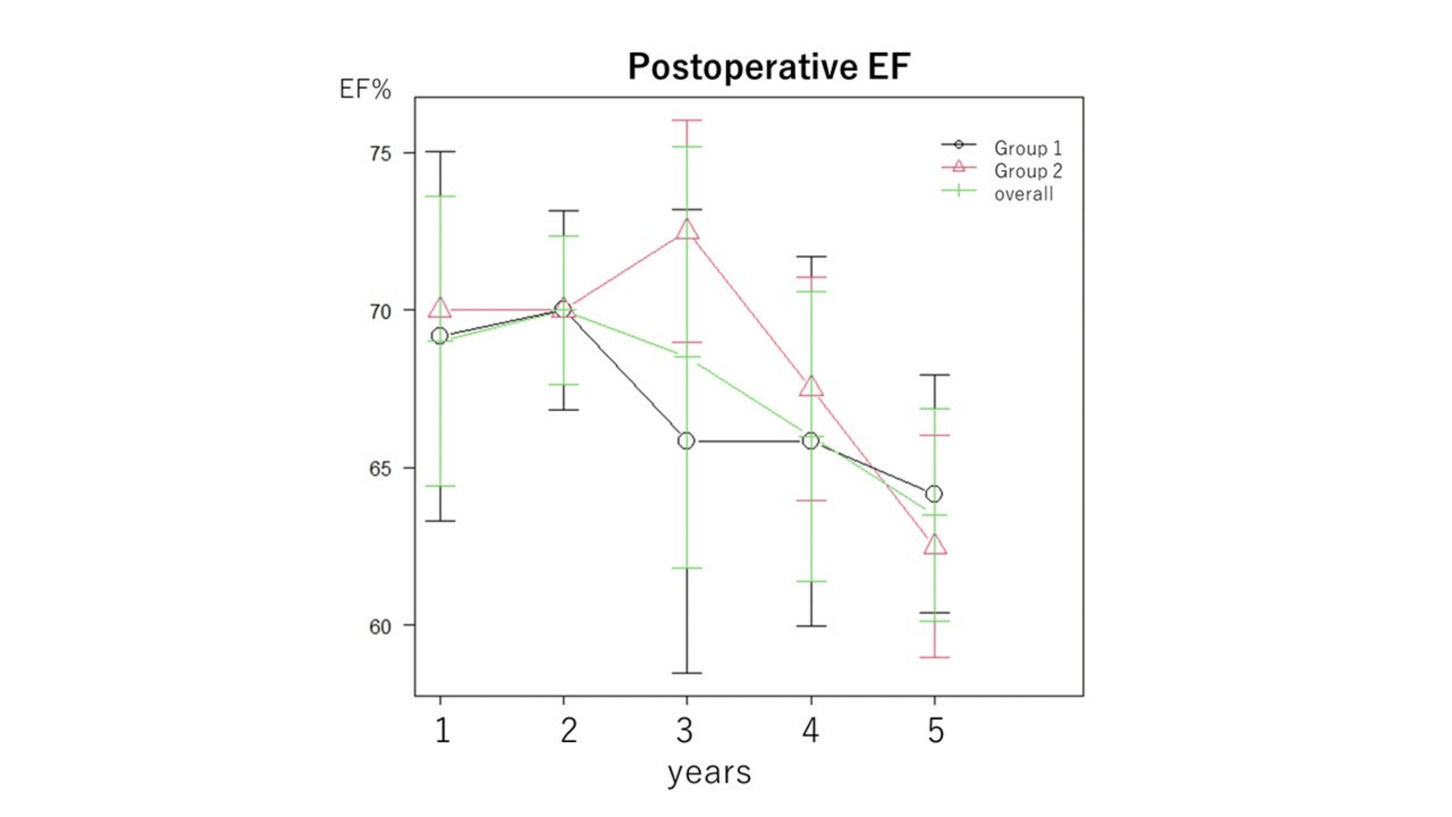
Postoperative EF. EF was assessed annually for five years after surgery. The values of each point in the line graph represent the median values. Groups 1 and 2 were tested using repeated-measures ANOVA. There were no significant differences between the groups. EF: ejection fraction; ANOVA: analysis of variance

**Figure 4:**
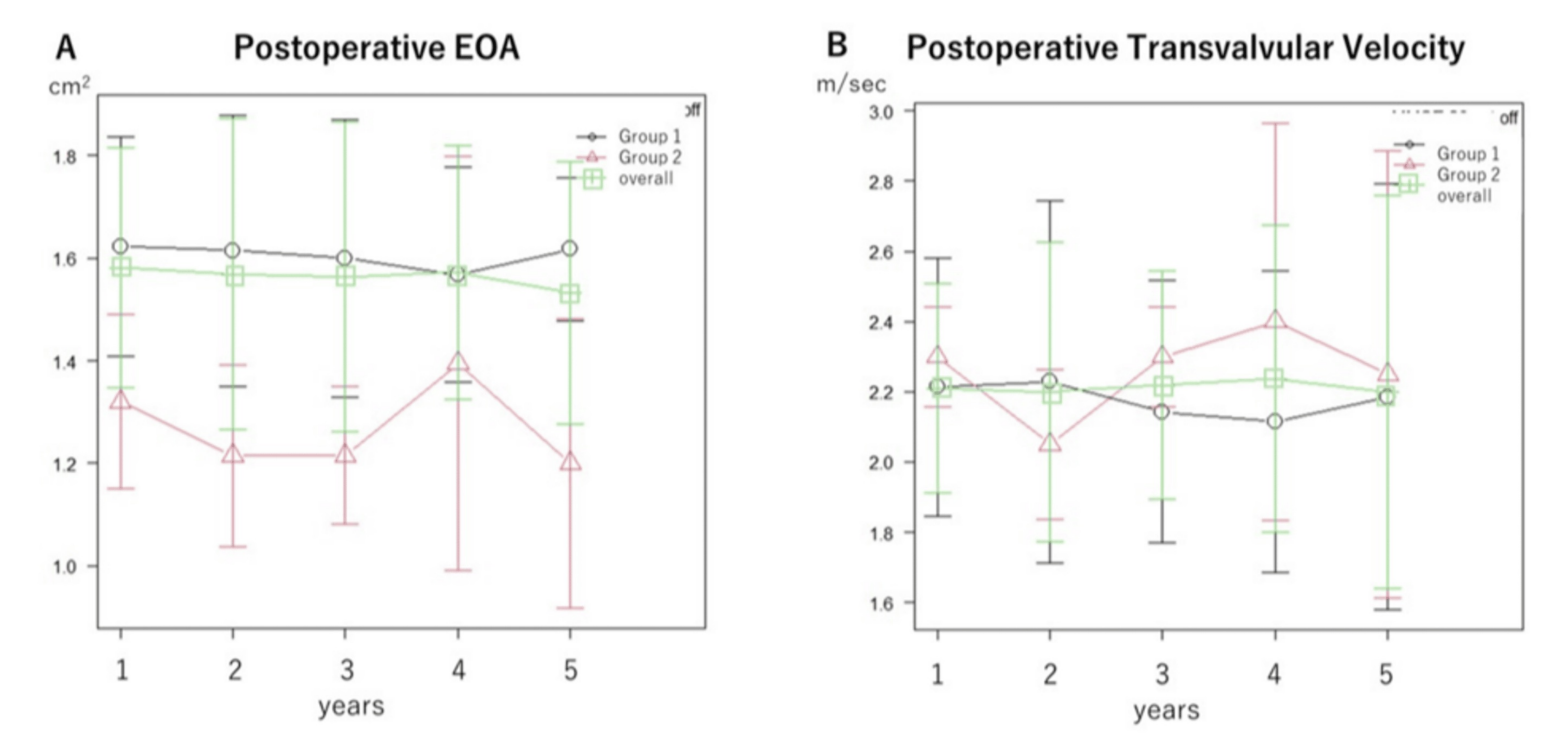
Postoperative EOA and transvalvular velocity. The EOA (A) and transvalvular velocity (B) were measured annually for five years after surgery. The values of each point in the line graph represent the median values. Groups 1 and 2 were tested using repeated-measures ANOVA. There were no significant differences between the groups. EOA: effective orifice area; ANOVA: analysis of variance

**Figure 5:**
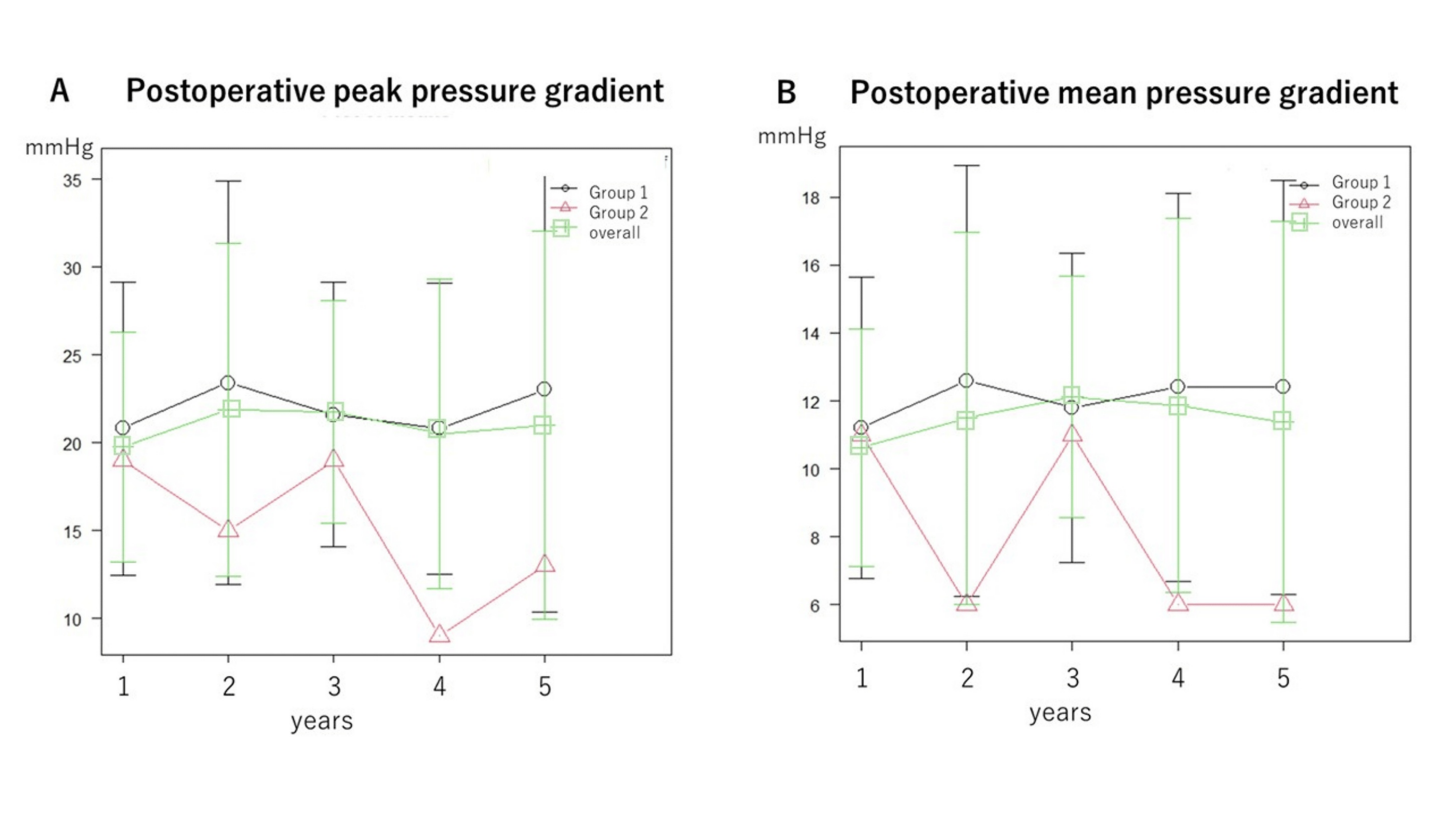
Postoperative peak and mean pressure gradients. Peak (A) and mean pressure (B) gradients were measured annually for five years after surgery. The values of each point in the line graph represent the median values. Groups 1 and 2 were tested using repeated-measures ANOVA. There were no significant differences between the groups. ANOVA: analysis of variance

**Figure 6:**
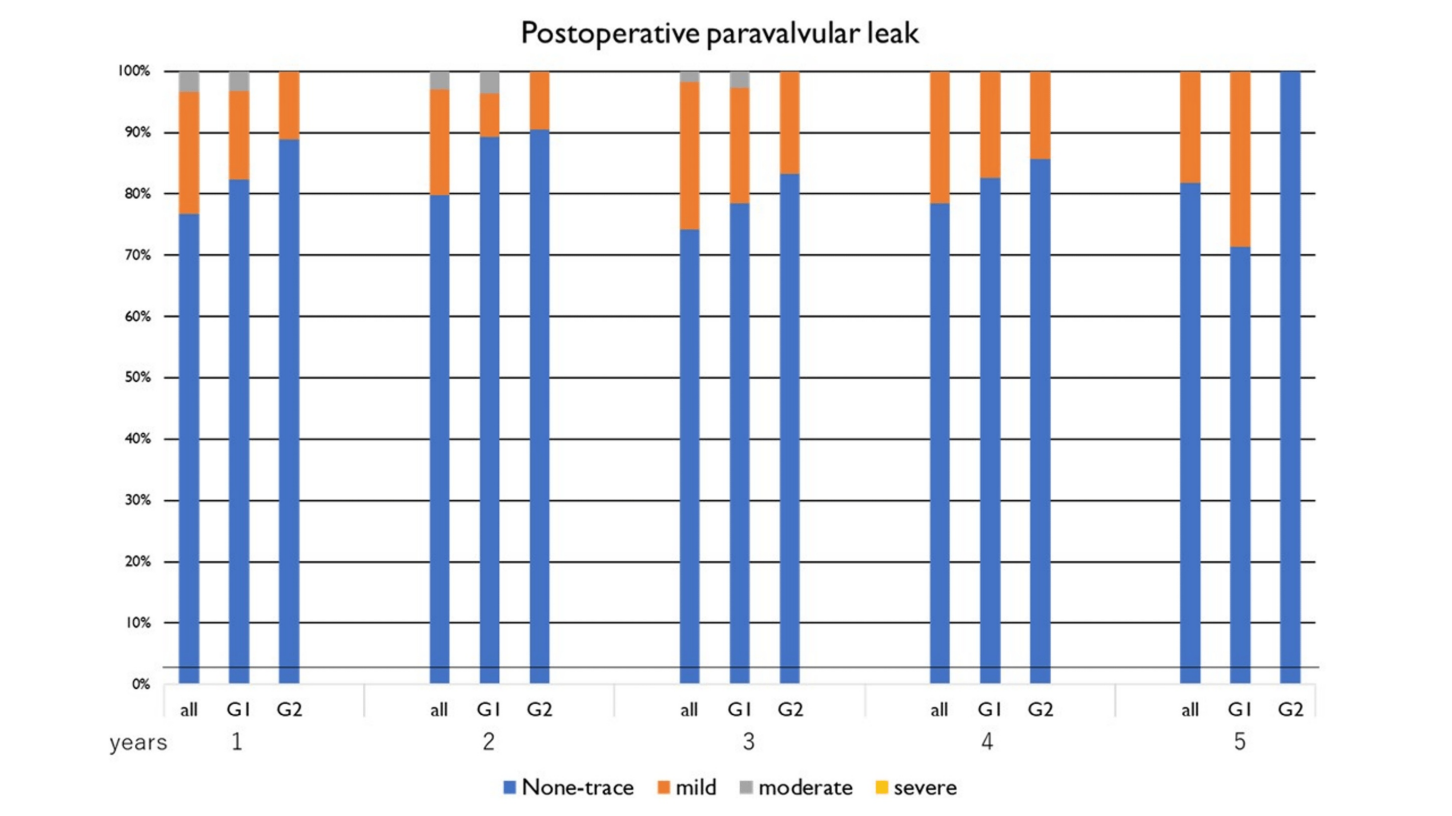
Postoperative paravalvular leakage. Paravalvular leakage was evaluated annually for five years after surgery. Paravalvular leakage was divided into two groups by severity: non-trivial-to-mild and moderate-to-severe. Groups 1 and 2 were assessed using Fisher’s exact test for each year. There were no significant differences between the groups in all years.

Echocardiography findings during the annual follow-up are shown in Figures 3-6. There were no significant differences between the two groups (Group 1 and 2) in EF (Figure 3), EOA (Figure 4A), transvalvular jet velocity (Figure 4B), mean and peak pressure gradient (Figures 5A, 5B), or paravalvular leakage (Figure 6).

## Discussion

Summary of results

This was a single-center, retrospective cohort study with a five-year follow-up of consecutive patients who underwent TAVI for severe AS. Our study demonstrated for the first time the association between cognitive function and risk of death in the aforementioned patients. Moreover, the relatively long-term postoperative follow-up is an advantage over other studies that had short or mid-term follow-up periods. We also showed the absence of differences in the preoperative patient background, procedural characteristics, pre- and postoperative prosthetic valve function, and left ventricular function.

Prognosis of TAVI patients in relation to preoperative cognitive function

Several studies have investigated factors that can predict the prognosis of patients with severe AS after TAVI [12,13]. Not surprisingly, in addition to procedural factors in TAVI, factors affecting the prognosis of most chronic cardiovascular diseases, such as renal function, sex, severity of heart failure, and anemia, were associated with the short-term prognosis of patients after TAVI [14,15]. We focused on cognitive function in this study because it is a critical issue considering the indication for TAVI and the postoperative prognosis of patients. TAVI is indicated for severe AS, for which AVR is not indicated. Therefore, it is often performed in elderly patients with high-risk comorbidities where the prevalence of cognitive impairment is high. Indeed, previous reports have shown that approximately 30-40% of patients with TAVI have cognitive decline, as often determined using the MMSE [15]. Similar to the results of these studies, cognitive decline was observed in approximately 32% of the patients using the MMSE in our study. In terms of prognosis, a recent study from Japan showed that cognitive impairment based on the MMSE score was an independent predictor of mortality one year after TAVI [15]. Interestingly, this study also showed that patients with cognitive impairment had higher non-cardiac mortality rates but not cardiac mortality, which is also consistent with our results. Saji et al. reported mid-term outcomes after TAVI [16]. They showed that patients with cognitive impairment, based on the Revised Hasegawa Dementia Scale, had a significantly higher mortality rate than those without impairment and that cognitive decline was an independent predictor of death. Our study further strengthens the argument that cognitive decline is present in more than a third of patients undergoing TAVI and is a significant prognostic issue at least five years postoperatively.

How does cognitive function affect the risk of death?

One of the advantages of our study over others was the continuous observation of left ventricular and prosthetic valve function over a five-year period. The fact that these indicators did not differ between the cognitively impaired and unimpaired groups, together with the findings on causes of death, suggests that higher mortality risk in the impaired group is probably due to non-cardiac reasons.

Based on our findings, we considered whether cognitive decline was only associated with high mortality or whether it directly increased mortality. Patients with cognitive decline are likely to have a worse prognosis than those without cognitive decline, even if they are the same age and have a similar clinical background because their aging progresses differently. It is possible that this aging is not measured as a variable. In other words, it may be an association mediated by unknown and unmeasurable confounding factors. However, cognitive decline may lead to poorer daily activity [10] and worsening health habits [17-19], such as poor adherence to regular medication, which also might increase the risk of adverse events and death. Ultimately, identifying the factors underlying the association between cognitive decline and mortality will require prospective studies that include not only biological factors but also socioeconomic factors such as caregiving.

Clinical perspectives

The results of our study, together with previous reports on the association between cognitive decline and adverse events [9,10,13-15], should be taken into account when considering indications for TAVI [8]. As successful TAVI itself, improved cardiac function, and uneventful prosthetic valves may not ultimately improve prognosis in patients with cognitive decline. Routine neurocognitive assessment is essential to identify extremely high-risk elderly patients when considering the indications for TAVI [8].

Limitations and strengths of our study

One strength of this study is that we used data from a single center. Thus, all TAVI procedures and techniques were performed by the same team, which could contribute to less biased outcomes. We followed up the patients based on their medical records via telephone survey. We were able to appropriately assess the patients’ long-term history. A limitation of this study was the small sample size; therefore, we adjusted HRs only by age because data were collected at a single center, and TAVI was only introduced when it was started at our facility. It is necessary to validate these results using larger-sized data samples that are prospectively collected from multiple centers. Another limitation of this study is that we were unable to assess the patients’ physical activity status continuously using the same modality. Between 2014 and 2016, we used instrumental ADL scores created by the Japanese Geriatric Society [20]. This score includes instrumental autonomy (getting on the bus, daily grocery shopping, meal preparation, bill payment, management of deposits and savings), intellectual activities (writing pension and other documents, reading newspapers, reading books, interests in health), and social roles (visiting friends, providing consultation to family or friends, visiting sick, talking to younger people). This is a subjective score; therefore, it may not reflect actual physical status. In 2016, we introduced the Short Physical Performance Battery [10,21], which is measured by physiotherapists and includes balance, gait speed, and chair stand tests. In 2018, we introduced the Barthel Index [22], which assesses 10 categories of daily living activities. These objective scoring tools that can accurately reflect patients’ physical activity and exercise tolerance status may be useful for future studies.

## Conclusions

Impaired cognition significantly and independently affected long-term outcomes in patients with severe AS undergoing TAVI. Considering the age of the patients in this study, the indications for TAVI should be carefully considered for each patient based on their cognitive function given that TAVI could be associated with complications that require surgical repair. Offering an opportunity to consider not to undergo TAVI may be suggested.
